# Patella-patellar tendon angle in relation to the medial patellar plica syndrome, chondromalacia patella, and infrapatellar fat pad syndrome

**DOI:** 10.1371/journal.pone.0265331

**Published:** 2022-03-17

**Authors:** Taeho Kim, Jin Kyem Kim, Hong Seon Lee, Dong Kyu Kim

**Affiliations:** Department of Radiology, The Armed Forces Capital Hospital, Seongnam, Korea; Assiut University Faculty of Medicine, EGYPT

## Abstract

The patella-patellar tendon angle (PPTA) assessing the sagittal patellar tilt was reported to be related with anterior knee pain. Herein, clinical effect of PPTA in patients with medial patellar plica (MPP) syndrome, chondromalacia patella, and infrapatellar fat pad (IPFP) syndrome, the most common causes of anterior knee pain, was evaluated. In this retrospective study, 156 patients with anterior knee pain who underwent magnetic resonance imaging (MRI) and arthroscopic surgery that confirmed isolated MPP syndrome, chondromalacia patella, or IPFP syndrome from June 2011 to January 2021 were included in the study group and 118 patients without knee pathology on MRI during the same period were included in the control group. The PPTA was measured on knee MRI and compared between the two groups. A receiver operating characteristic (ROC) analysis was used to evaluate the value of PPTA for predicting the risk of patellofemoral joint disorder. The mean PPTA was significantly smaller in study group (138.1 ± 4.2°) than control group (142.1 ± 4.3°) (*p* < 0.001). However, there was no significant difference in PPTA among the patients with MPP syndrome, chondromalacia patella, and IPFP syndrome. Furthermore, the ROC analysis revealed that the area under curve, sensitivity, and specificity for predicting the risk of patellofemoral joint disorders were 0.696, 70.3% and 57.6%, respectively, at a PPTA cutoff of 138.3°. Therefore, the smaller PPTA may be associated with MPP syndrome, chondromalacia patella, and IPFP syndrome. Furthermore, PPTA could be a predictive factor for the risk of patellofemoral joint disease in patients with anterior knee pain.

## Introduction

Anterior knee pain (AKP) is a common clinical symptom, accounting for 11–17% of physician visits for knee symptoms, but the diagnosis of AKP is often challenging [1, 2]. Although the causes of AKP vary, medial patellar plica (MPP) syndrome, chondromalacia patella, and infrapatellar fat pad (IPFP) syndrome are well-known causes of chronic AKP [3, 4]. MPP syndrome and IPFP syndrome could be diagnosed based on clinical symptom, physical examination, and MRI, but the accurate diagnosis could also require arthroscopic findings [5–8]. Chondromalacia patella could be diagnosed accurately on MRI, but it also has been shown to underdiagnose the grade and size of chondral lesion compared with arthroscopy [9, 10].

In recent studies, sagittal plane tilting deformity of the patellofemoral joint has been described. Sagittal patellar tilt is assessed by measuring the patella-patellar tendon angle (PPTA) [3]. Some previous studies reported that patients with MPP syndrome, chondromalacia patella, and IPFP syndrome had smaller PPTA than healthy individuals [3, 4]. However, there is limited published information whether there is a difference in PPTA according to each different patellofemoral joint disorder. Furthermore, there is no established normal value of PPTA.

Therefore, the purposes of our study are: 1) to evaluate sagittal patellar tilt in patients with MPP syndrome, chondromalacia patella, and IPFP syndrome by measuring PPTA; 2) to compare the PPTA values among the patients with different disorders; and 3) to determine the PPTA cutoff value to predict the risk of patellofemoral joint disease.

## Materials and methods

### Study population

This single-center retrospective study was approved by the Institutional Review Board of Armed Forces Capital Hospital with a waiver of informed consent (IRB approval number: AFCH-20-IRB-030).

From June 2011 to January 2021, patients who underwent knee MRI due to anterior knee pain (n = 4118) were identified. Among them, patients diagnosed as isolated MPP syndrome, chondromalacia patella, and IPFP syndrome based on both MRI and arthroscopic findings were included in the study group (Group A). However, patients with 1) any history of knee surgery, 2) meniscal pathology, 3) inflammatory arthritis, 4) ligament injury, 4) space occupying lesion, 5) patellofemoral malalignment in the horizontal plane (patellar tilt or subluxation), and 6) sagittal plane malpositioning pathology (patella alta or patella baja) were excluded. The control group (Group B) consisted of patients who complained subjective anterior knee pain, but had no diagnosed pathology on MRI.

### MRI protocol

MRI examinations were performed using 1.5-T (Signa Explorer, GE Healthcare, USA) or 3.0-T (Discovery MR 750w, GE Healthcare, U.S.A.) MR scanners with the unenhanced knee protocol. The standard protocol of 1.5-T MRI consisted of axial T2-weighted fat saturation (repetition time (TR)/echo time (TE) 3462/38 ms, 3-mm slice thickness), coronal T1-weighted (TR/TE 476/6 ms, 3.5-mm slice thickness) and T2-weighted fat saturation (TR/TE 5500/63 ms, 3.5-mm slice thickness), and sagittal proton density-weighted (TR/TE 2200/40 ms, 3.5-mm slice thickness) and T2-weighted fat saturation (TR/TE 2627/41 ms, 3.5-mm slice thickness) sequences through the entire knee using a 32-channel body coil. The field of view (FOV) for each sequence was 18 × 18 cm with a 384 × 256 matrix. The protocol of 3.0-T MRI consisted of axial T2-weighted fat saturation (TR/TE 3424/40 ms, 3-mm slice thickness), coronal T1-weighted (TR/TE 728/8 ms, 3.5-mm slice thickness) and T2-weighted fat saturation (TR/TE 4162/59 ms, 3.5-mm slice thickness), and sagittal proton density-weighted (TR/TE 2156/41 ms, 3.5-mm slice thickness) and T2-weighted fat saturation (TR/TE 4018/37 ms, 3.5-mm slice thickness) sequences using a 23-channel body coil with a FOV of 18 × 18 cm and 384 × 288 acquisition matrix.

### Image analysis

The PPTA was obtained by measuring the angle between a line connecting the patellar upper and lower poles and a line from the inferior patella to the tibial tuberosity [3]. To evaluate the PPTA values in each patient, sagittal proton density-weighted images on MRI were retrospectively and independently reviewed by two radiologists with 6 and 11 years of radiology experience, respectively, who were blinded to any clinical patient information. Thus, at the time of measurement, the patients’ grouping was unknown.

### Clinical records

Clinical information including age, gender, body mass index (BMI), affected side of knee (right vs left), and interval periods from the MRI examination to arthroscopic surgery was collected from the electronic medical charts. Subjective pain was evaluated with the use of the numeric rating scale (NRS) at the time of the clinical visit.

### Statistical analysis

Continuous variables are expressed as mean ± standard deviation (SD). Continuous variables were compared by using independent t-tests or one-way analysis of variance, while categorical variables were compared by using x^2^ or Fisher exact tests.

Intraobserver and interobserver agreement regarding PPTA was evaluated by intraclass correlation coefficients (ICCs). ICC results were interpreted according to the following criteria: poor (ICC < 0.50), moderate (0.50 < ICC < 0.75), good (0.75 < ICC < 0.90), and excellent (ICC > 0.90) [11]. A receiver operating characteristic (ROC) analysis was conducted to assess the performance of PPTA for the prediction of the risk of knee patellofemoral joint disorder, based on the values of sensitivity, specificity, and area under curve (AUC). The optimal cut-off value was determined to maximize the sum of sensitivity and specificity.

All statistical analyses were performed with R software (version 3.3.3; R Development Core Team, R Foundation for Statistical Computing, Vienna, Austria) and *p* values < 0.05 were considered statistically significant.

## Results

### Patients

Between June 2011 and January 2021, a total of 156 patients were included in the study group (Group A): 86 patients with MPP syndrome, 44 patients with chondromalacia patella, and 26 patients with IPFP syndrome. The control group (Group B) consisted of 118 patients without any diagnosed knee pathology on MRI. Among the patients in control group, 13 patients underwent diagnostic arthroscopy following MRI, but also had no diagnosed pathology on arthroscopic finding.

### Patella-patellar tendon angle differences between study and control group

The baseline characteristics of the patients are summarized in Table 1. There was no significant difference between study group and control group in age (30.1 ± 7.8 years vs. 30.2 ± 4.1 years, *p* = 0.911), gender (male: female; 134:22 vs. 106:12, *p* = 0.328), BMI (21.5 ± 2.9 vs. 22.2 ± 2.7, *p* = 0.514), affected side of knee (right: left; 87:69 vs. 69:54, *p* = 0.801), and interval periods from the MRI examination to arthroscopic surgery (49.2 ± 13.7 days vs. 42.7 ± 21.1 days, *p* = 0.543).

**Table 1:** Baseline characteristics of patients.

	Group A (n = 156)	Group B (n = 118)	P value	Total (n = 274)
Age (years)*	30.1 ± 7.8	30.2 ± 4.1	0.911	30.1 ± 6.7
BMI (kg/m^2^)	21.5 ± 2.9	22.2 ± 2.7	0.514	21.8 ± 2.8
Gender, *n* (%)			0.328	
Male	134 (85.9)	106 (89.8)		240 (87.6)
Female	22 (14.1)	12 (10.2)		34 (12.4)
Side, *n* (%)			0.801	
Right	87 (55.8)	64 (54.2)		151 (55.1)
Left	69 (44.2)	54 (45.8)		123 (44.9)
NRS	3.1 ± 0.7	2.0 ± 0.6	< 0.001	2.6 ± 0.9
Time interval (days)**	49.2 ± 13.7	42.7 ± 21.1	0.543	46.3 ± 14.9
PPTA (°)	138.1 ± 4.2°	142.1 ± 4.3°	< 0.001	140.0 ± 4.3°

Group A: 156 patients with MPP syndrome (n = 86), chondromalacia patella (n = 44) or IPFP syndrome (n = 26), Group B: 118 patients without any diagnosed knee pathology

BMI = body mass index, NRS = numeral rating scale, PPTA = patella-patellar tendon angle

*Results of continuous values are expressed as the mean ± standard deviation and categorical values are expressed as numbers of patients with percentages

**Time interval: interval periods from the MRI examination to arthroscopic surgery.

**Fig 1:**
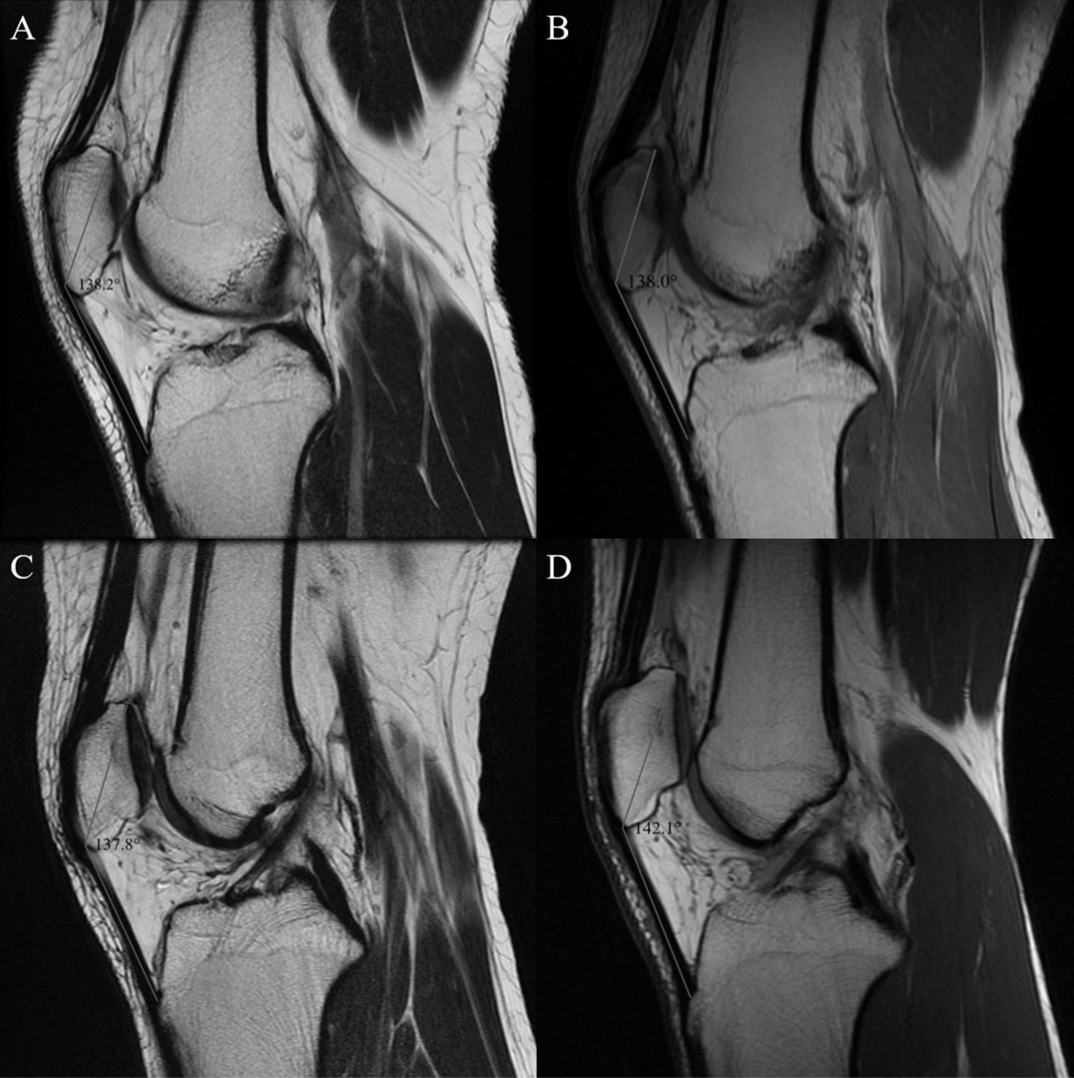
Measurements and comparison of patella-patellar tendon angle (PPTA) in patients who complained anterior knee pain. In study group, measured PPTA on sagittal proton density-weighted images was (a) 138.2° in a 21-year-old man with medial patellar plica syndrome, (b) 138.0° in a 22-year-old man with chondromalacia patella, and (c) 137.8° in a 21-year-old man with infrapatellar fat pad syndrome. (d) However, in a 22-year-old man with no abnormality on MRI and diagnostic arthroscopy, the PPTA was 142.1° which was significantly higher than that of patients in study group.

However, the mean NRS was significantly higher in study group than control group (3.1 ± 0.7 vs. 2.0 ± 0.6, *p* < 0.001) and mean value of PPTA was significantly lower in study group than control group (138.1 ± 4.2° vs. 142.1 ± 4.3°, *p* < 0.001) (Fig 1).

**Fig 2:**
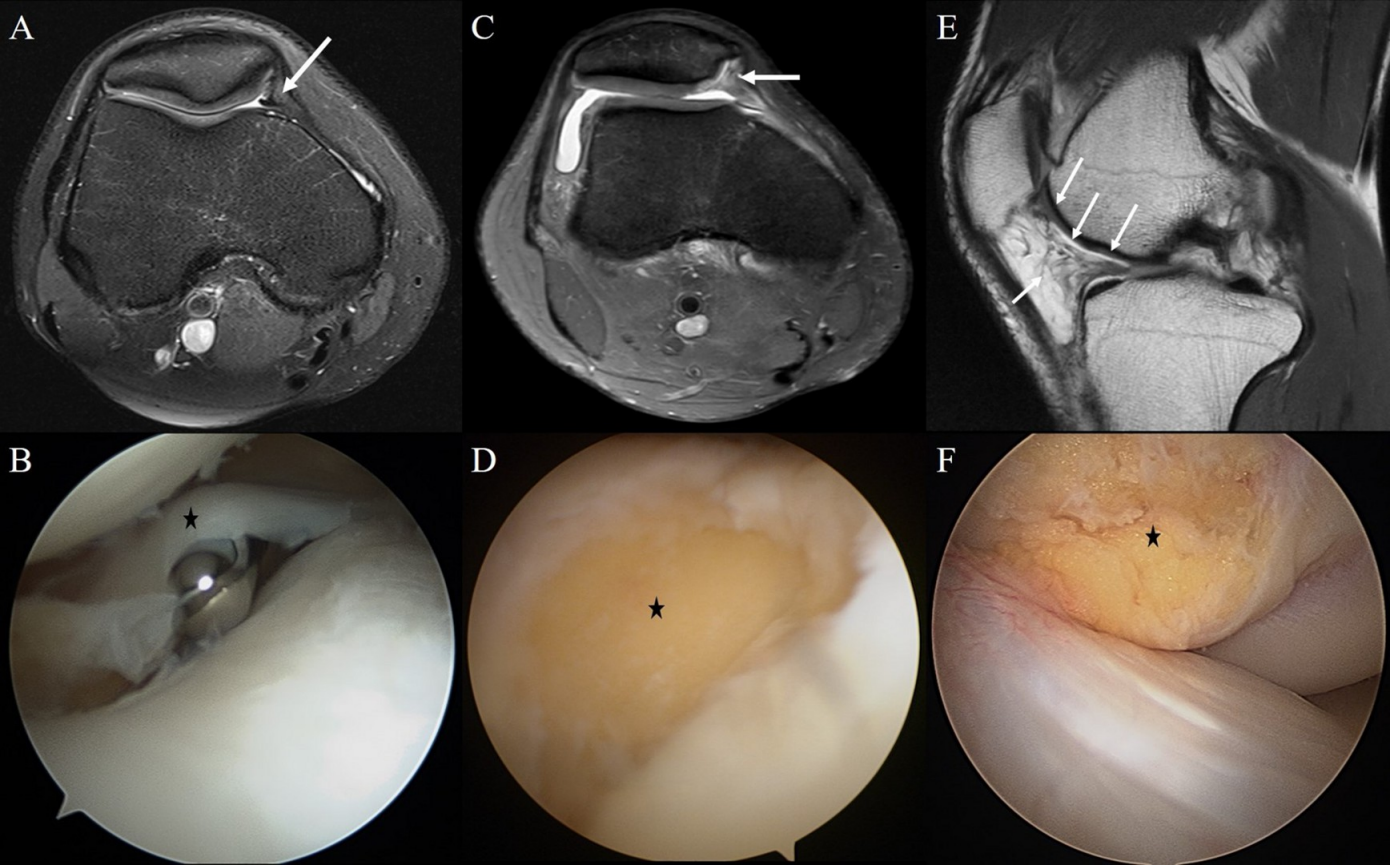
Knee MRI and arthroscopic findings of patients in study group. (a, b) A 21-year-old man with medial patellar plica (MPP) syndrome showed thickened MPP (arrow) and impingement MPP (asterisk) between medial femoral condyle and patella. On MRI, the PPTA was 138.2°. (c, d) A 22-year-old man with chondromalacia patella showed contour defects in the cartilage surface of patella medial facet (arrow) and there was 1.5 ⅹ 1.5 cm sized cartilage defect (asterisk) in that area on arthroscopy. The PPTA was 138.0° in this patient. (e, f) A 21-year-old man with infrapatellar fat pad (IPFP) syndrome showed soft tissue infiltrations (arrows) at IPFP on MRI and fat tissue hypertrophy (asterisk) on arthroscopy. The PPTA was 137.8°.

ROC analysis revealed that PPTA lower than 138.3° represented a potential cutoff value for the prediction of the risk of knee patellofemoral joint disorder with a sensitivity of 70.3% and a specificity of 57.6% (AUC 0.696, 95% CI 0.635–0.746, *p* < 0.001) (Fig 2).

The intraobserver and interobserver agreement for PPTA in all patients including study and control group was 0.902 (95% CI 0.849–0.955) and 0.816 (95% CI 0.735–0.872), respectively.

### Patella-patellar tendon angle differences among the patients with MPP syndrome, patellar chondromalacia and IPFP syndrome

**Fig 3:**
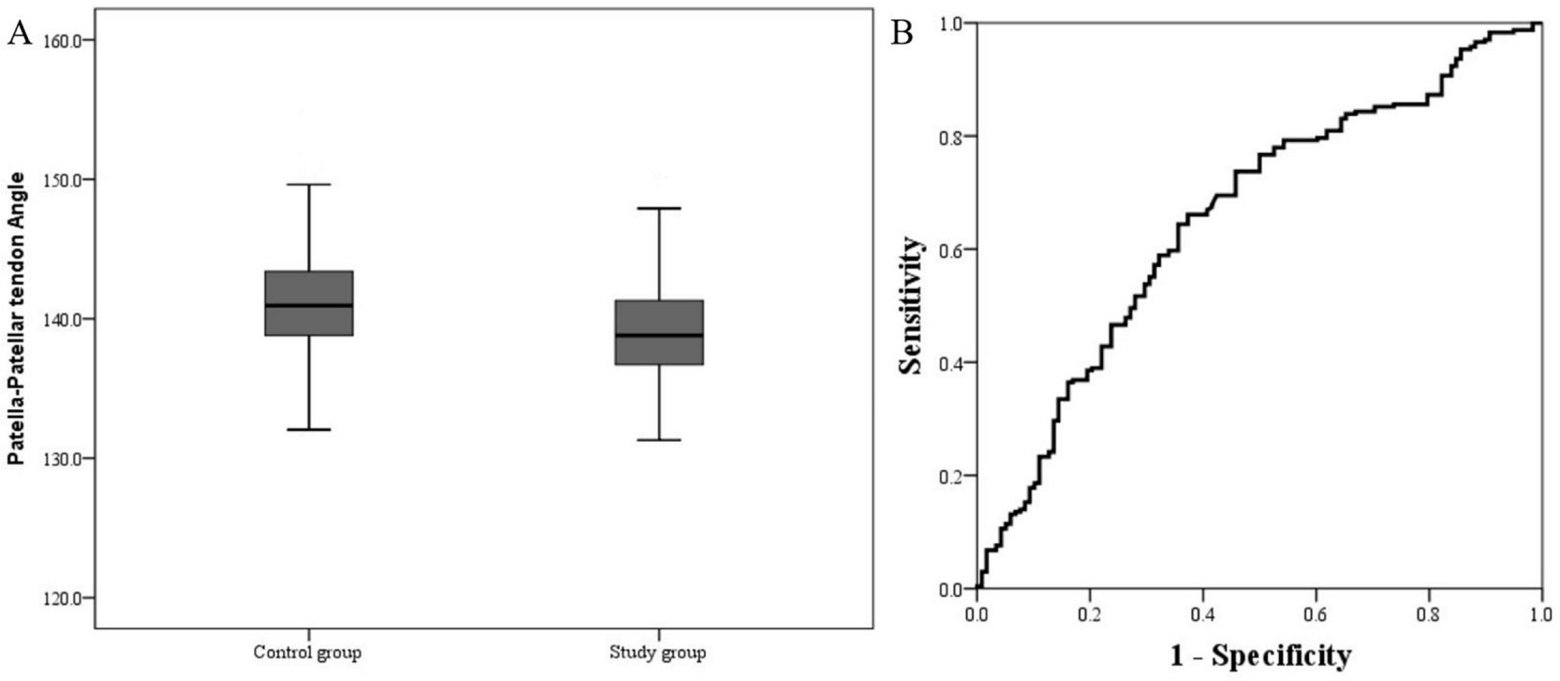
Comparison of PPTA between the two groups (study group vs. control group) and receiver operating characteristic (ROC) analysis to assess the performance of PPTA for the prediction of the risk of knee patellofemoral joint disorder. (a) Box-plot showed mean PPTA was significantly lower in study group than control group (138.1 ± 4.2° vs. 142.1 ± 4.3°, p < 0.001). (b) The ROC curve of PPTA for predicting the patellofemoral joint disorder showed the AUC of 0.696 at a cutoff value of 138.3°, with a sensitivity of 70.3% and a specificity of 57.6%.

Of the 156 patients in study group, 86 patients suffered MPP syndrome, 44 patients suffered chondromalacia patella, and 26 patients suffered IPFP syndrome (Fig 3). There was no significant difference not only in age, gender, BMI, affected side of knee, NRS, interval periods from the MRI examination to arthroscopic surgery, but also in PPTA (MPP syndrome: 138.1 ± 4.3° vs. Chondromalacia patella: 138.4 ± 4.2° vs. IPFP syndrome: 137.6 ± 4.3°, *p* = 0.406) (Table 2).

**Table 2:** Comparison of baseline characteristics of patients in study group.

	MPP syndrome (n = 86)	Chondromalacia patella (n = 44)	IPFP syndrome (n = 26)	P value*
Age (years)*	29.2 ± 5.6	31.5 ± 9.1	30.8 ± 6.6	0.416
BMI (kg/m^2^)	21.4 ± 2.9	21.7 ± 3.0	21.5 ± 2.7	0.512
Gender, *n* (%)				0.687
Male	72 (83.7)	39 (88.6)	23 (88.5)	
Female	14 (16.3)	5 (11.4)	3 (11.5)	
Side, *n* (%)				0.209
Right	53 (61.6)	20 (45.5)	14 (53.8)	
Left	33 (38.4)	24 (54.5)	12 (46.2)	
NRS	3.1 ± 0.4	3.1 ± 0.5	3.2 ± 0.4	0.151
Time interval (days)**	48.2 ± 13.3	53.3 ± 11.8	45.4 ± 17.4	0.231
PPTA (°)	138.1 ± 4.3	138.4 ± 4.2	137.6 ± 4.3	0.406

BMI = body mass index, MPP = medial patella plica, IPFP = infrapatellar fat pad, NRS = numeral rating scale, PPTA = patella-patellar tendon angle

*Comparison among the three groups (MPP syndrome vs Chondromalacia patella vs IPFP syndrome) was performed with the one-way analysis of variance for continuous variables and the x2 or Fisher exact test for categorical variables.

**Time interval: interval periods from the MRI examination to arthroscopic surgery.

## Discussion

In the present study, sagittal patellar tilt was evaluated in patients with MPP syndrome, chondromalacia patella, and IPFP syndrome by measuring PPTA. This study demonstrated that PPTA was significantly lower in patients with MPP syndrome, chondromalacia patella, and IPFP syndrome than in those of control group, which was consistent with the results of previous studies [3, 4]. However, there was no significant difference in PPTA among the patients in each disease. Furthermore, ROC analysis revealed that a PPTA ≤ 138.3° could serve as a predictive factor for patellofemoral joint disorders.

The most known reasons of AKP are trauma and overuse [12, 13], but MPP syndrome, chondromalacia patella, and IPFP syndrome are well-known conditions that could cause AKP [3, 4]. The medial, lateral, superior and inferior synovial plicae are normal structures of the knee joint, but plica-related AKP is mostly caused by impingement of medial plica. If there are pathogenic factors including overuse injury, trauma or inflammatory arthropathy, the inflammatory reaction causes thick and hardened MPP by fibrosis, resulting entrapment of MPP between medial femoral condyle and patella, and finally leading to AKP [14, 15]. Infrapatellar fat pad also undergoes fibrosis or inflammatory hypertrophy as a result of trauma or irritation, and it causes AKP [6, 7]. In these conditions, combination of smaller PPTA could aggravate the AKP by supraphysiologic loading on patellofemoral joint. Furthermore, smaller PPTA itself could be an intrinsic factor for AKP. Some previous studies reported pathologic loading on patellofemoral joint by smaller PPTA was one of the etiologic factors for chondromalacia patella [3, 16, 17]. The breakdown and softening of cartilage at the underside of patella, which is termed chondromalacia patella, leads to AKP when the patella and femur rub together.

Currently, the diagnosis of MPP syndrome and IPFP syndrome is based on medical history taking, physical examination, MRI findings, and arthroscopic findings. However, there is no established diagnostic modality for these diseases. Thus, the diagnosis is usually difficult and diagnostic accuracy may vary according to the clinicians’ experience [14, 15, 18, 19]. Therefore, the cutoff value of PPTA ≤ 138.3° from this study could be worthwhile in that it could support more accurate diagnosis of MPP syndrome and IPFP syndrome. In addition, in patients with chondromalacia patella, it could be a predictive factor that chondromalacia and AKP may worsen when PPTA is smaller than the cutoff value. However, the sensitivity, specificity, and AUC of the cutoff value is relatively lower (sensitivity: 70.3%, specificity: 57.6%, AUC: 0.696) to be used generally. Furthermore, to the best of our knowledge, there was no previous study to determine the PPTA cutoff value in the disease groups and some previous studies reported the normal mean PPTA value as 145.1° [3, 20]. Further studies are needed to determine the normal value and statistically significant cutoff value of PPTA.

There were some limitations in our study. The first was its retrospective design and inherent selection bias, which could have affected the results. Second, the PPTA could be changed at different knee flexion angles during the MRI examination. However, knee MRI scanning was performed with knee extension in each patient at our hospital and knee extension would be maintained during MRI examination considering the knee position. Third, although all 156 patients in study group underwent arthroscopic surgery following MRI, only 13 patients underwent arthroscopy in control group. For determining the normal value of PPTA, arthroscopic confirmation that there was no pathologic finding in the knee joint may be needed. In this study, there was no specific arthroscopic findings in these 13 patients of control group. Further study of large sample size with arthroscopic finding may provide the statistically significant cutoff value of PPTA in disease group compared with healthy individuals. Finally, a previous study in patients with chondromalacia patella, PPTA values were compared by classifying the lesion location as superior, middle, and inferior zones. In that study, PPTA was lower in patients with superior and inferior patella chondromalacia than those in the control group [3]. However, in our study, the number of patients with chondromalacia patella (n = 44) was relatively small and most of the patients had chondromalacia at superior and middle zones (superior, n = 26; middle, n = 16; inferior, n = 2). Thus, chondromalacia location could not be divided in this study. However, we obtained meaningful results that PPTA was significantly lower in patients with chondromalacia than in those of control group, but there was no significant difference in PPTA among the patients with chondromalacia patella, MPP syndrome, and IPFP syndrome.

In conclusion, the PPTA was significantly lower in patients with MPP syndrome, chondromalacia patella, and IPFP syndrome than in those who had AKP, but without diagnosed knee pathology. However, there was no significant difference in PPTA among the patients with MPP syndrome, chondromalacia patella, and IPFP syndrome. In addition, the present study showed PPTA smaller than 138.3° could be a predictive factor for patellofemoral joint disorder in patients with AKP. Therefore, evaluation of sagittal patellar tilt by measuring PPTA could have clinical value for diagnosis of patellofemoral joint disorder in patients with AKP.
